# Variant analysis of human papillomavirus type 52 in Iranian women during 2018−2020: A case‐control study

**DOI:** 10.1002/hsr2.2158

**Published:** 2024-07-01

**Authors:** Parvin Jalali‐Alhosseini, Zabihollah Shoja, Somayeh Jalilvand

**Affiliations:** ^1^ Department of Virology, School of Public Health Tehran University of Medical Sciences Tehran Iran; ^2^ Department of Virology Pasteur Institute of Iran Tehran Iran

**Keywords:** cervical cancer, human papillomavirus, lineage, sublineage, type 52

## Abstract

**Background and Aims:**

Knowing the regional variants of distinct human papillomavirus (HPV) types is valuable as it can be beneficial for studying their epidemiology, pathogenicity, and evolution. For this reason, the sequence variations of the E6 gene of HPV 52 were investigated among women with normal cervical cytology and premalignant/malignant cervical samples.

**Methods:**

Sixty‐four HPV 52‐positive samples were analyzed using semi‐nested PCR and sequencing.

**Results:**

Our findings showed that all samples belonged to lineage A (61%) or B (39%). Among samples that were infected with the A lineage, sublineages A1 and A2 were detected and sublineage A1 was dominant. No association was found between lineages and stage of disease (*p* > 0.05).

**Conclusion:**

Our results revealed that the A lineage, sublineage A1, and B lineage were common in Iranian women. Nevertheless, more studies with larger sample sizes are required to estimate the pathogenicity risk of HPV 52 lineages in Iranian women with cervical cancer.

## 1 INTRODUCTION

Papillomaviruses are small double‐stranded DNA viruses that are considered the etiological agent of different kinds of warts and several cancers including cervical, vulvar, vagina, penile, anal, head, and neck.^1^, ^2^, ^3^, ^4^ More than 200 different human papillomavirus (HPV) types are reported; among which 40 types can infect the anogenital areas.^2^ However, 14 HPV types are designated as high‐risk (HR)‐HPV types, including HPV 16, 18, 31, 33, 35, 39, 45, 51, 52, 56, 58, 59, 66, and 68 that can persist and cause several malignancies, particularly cervical cancer.^5^, ^6^, ^7^, ^8^, ^9^

Although HPV infections mostly resolve within 6 months up to 2 years after acquisition, a small proportion of infections with HR‐HPV types can persist and consequently lead to cervical cancer. The global prevalence of the 10 most common HPV types was reported to be 55.4%, 16.1%, 4.7%, 4.1%, 3.8%, 3%, 2.8%, 1.9%, 1.2%, and 0.9% for HPV 16, 18, 45, 33, 31, 58, 52, 35, 59, and 56 in cervical cancer, respectively.^10^ The five most frequent HPV types are HPV 16 (53%), 18 (15%), 31 (4%), 33 (3%), and 45 (3%) in Iranian women with cervical cancer.^11^

A distinct HPV type is one where the DNA sequence of L1 is at least 10% different from that of any other characterized type. Lineage and sublineage are designated for those HPVs within a given type that differ by 1%−10% and 0.5%−1% across the genome, respectively. HPV 52 includes four distinct lineages, A, B, C, and D, and six different sublineages, including A1, A2, B1, B2, C1, and C2.^12^

While the geographic associations for the distinct lineages and sublineages of HPV 16 and 18 are well‐known,^5^, ^13^, ^14^, ^15^, ^16^ the geographic associations of other types are less known. To address an association between the different lineages and sublineages of HPV 52 and ethnicity, further studies are required in the world. Some mutation types in the same gene show diverse carcinogenic risks in different countries and regions. Indeed, HPV16 E6 variant analysis shows that G350 (L83V) change is the most common mutation in Europe and America whereas G178 (D25E) substitution is predominantly observed in East Asia. Therefore, it is suggested that mutations at some sites may change the risk of carcinogenesis and increase the escape of the host immune system.^17^

Although the distribution of HPV types is well‐known in Iran,^11^ there is much less identified about HPV lineages and sublineages in this country.^15^, ^18^, ^19^, ^20^, ^21^ The data in this regard is important as it would provide a rationale for future studies on their epidemiology, evolution, pathogenicity, and biology. In agreement with this, the present study intended to investigate the sequence variations of the E6 gene to identify the common HPV 52 lineages and sublineages circulating in Iran.

## 2 MATERIALS AND METHODS

### 2.1 Study population

To investigate the lineages and sublineages of HPV 52, a case‐control study was conducted from 2018 to 2020. One‐hundred and forty‐two formalin‐fixed paraffin‐embedded (FFPE) specimens (98 invasive cervical cancer and 44 premalignant cervical lesions) were collected from Immam‐Khomeini Hospital in Tehran. One‐hundred and thirty‐five ThinPrep Pap Test specimens that were positive as a pooled of 12 HR‐HPVs (31, 33, 35, 39, 45, 51, 52, 56, 58, 59, 66, and 68) by Cobas assay and 49 ThinPrep Pap Test samples that were positive for HPV 52 (previously genotyped using INNO‐LiPA® HPV Genotyping assay) were obtained from several referral laboratories in Tehran.

All subjects of this study signed the informed consent that was approved by the local ethical committee of Tehran University of Medical Sciences (IR.TUMS.SPH.REC.1399.253). The demographic data were extracted from their medical records.

### 2.2 Lineage and sublineage analysis of HPV 52

According to the manufacturer's instructions, DNA was isolated from ThinPrep Pap Test samples using the High Pure Viral Nucleic Acid Kit (Roche Diagnostics GmbH; Roche Applied Science). DNA from FFPE specimens was extracted by phenol–chloroform assay according to an earlier published procedure.^22^

The HPV genome was amplified in 142 FFPE specimens by nested PCR with MY09/MY11 and GP5^+^/GP6^+^ primer pairs to obtain a 150 bp amplicon of the L1 gene. All HPV‐positive specimens were sequenced using BigDye® Terminator v3.1 Cycle Sequencing Kit and a 3130 Genetic Analyzer Automated Sequencer as specified by Applied Biosystems manuals. All obtained sequences were edited by Bioedit software and blasted using the Blast server (http://www.ncbi.nlm.nih.gov/blast/) to know HPV genotypes. All 135 samples that were positive as the pooled of 12 HR‐HPVs using Cobas assay were screened by nested‐PCR to amplify of E6 gene of HPV 52 as described below.

The complete E6 gene of HPV 52 (nucleotide [nt] 102−548) was analyzed by semi‐nested PCR with the following primers: TAACCGAAAACGGTCAGA (52E6‐F), GTTTCAGGTTGCAGATCTAAT (52E6‐R1), and TTGCTTTGTCTCCACGCATGAC (52E6‐R2). The PCR reaction was carried out in a 50 μL reaction mixture including 1.5 mM MgCl_2_, 50 μM of each dNTP, 10 pmol of each primer, 2 U of Taq DNA polymerase, and 100−200 ng of DNA template (the first round) and 1 μL of the first round (the second round). PCR amplification cycles were as follows: 35 cycles of 95°C for 30 s, 52°C for 50 s, and 72°C for 50 s (the first round) and 35 cycles of 95°C for 20 s, 52°C for 40 s, and 72°C for 40 s (the second round). As a negative control, a reaction mixture lacking template DNA was included in every set of PCR runs.

To analyze HPV 52 variants, the PCR products were sequenced using bidirectional direct sequencing as described above. All sequences are submitted to GenBank and are available with the accession numbers OL627421‐OL627480.

To identify HPV 52 lineages and sublineages, the sequences of all studied samples were aligned to reference sequences including X74481 (A1), HQ537739 (A2), HQ537740 (B1), HQ537743 (B2), HQ537744 (C1), HQ537746 (C2), and HQ537748 (D),^12^ as well as more sequences that were obtained from http://www.ncbi.nlm.nih.gov/. The phylogenetic tree was constructed by the maximum likelihood method using Mega software version 11. The reliability of the phylogenetic tree was calculated by the measurement of bootstrap with 1000 replicates.

### 2.3 Statistical analysis

The statistical analysis was executed by the Mantel−Haenszel *χ* ^2^ test (two‐sided) using Epi Info 7; Statistical Analysis System Software. The *p*‐value was considered statistically significant when it was less than 0.05.

## 3 RESULTS

HPV 52 was found in three out of 142 FFPE specimens (2.1%) and all of them were obtained from women with invasive cervical cancer. In 135 samples that were positive by Cobas assay, eight specimens (5.9%) were infected with HPV 52. In total, 64 samples, including 44 normal and 20 premalignant/malignant cervical samples, were HPV 52‐positive and investigated for determining lineages and sublineages of HPV 52.

**Figure 1:**
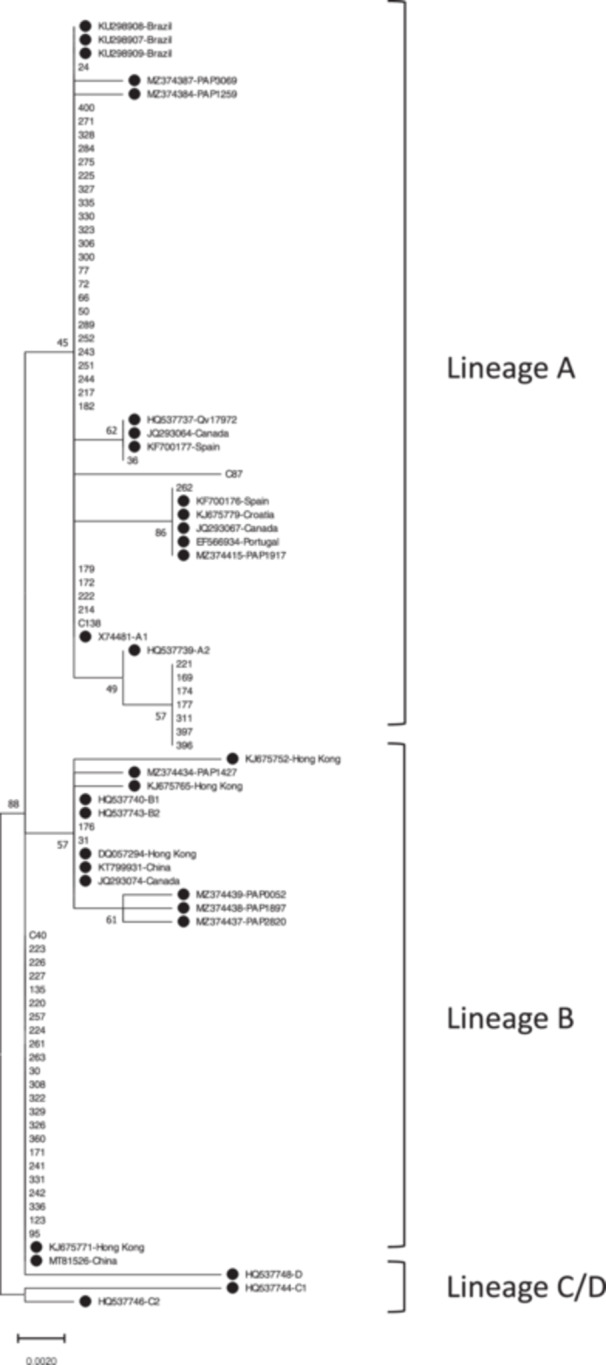
Phylogenetic analysis of the HPV 52 E6 gene was conducted in MEGA11 by the Maximum Likelihood method based on the Tamura 3‐parameter model. The reference sequences were indicated by a black circle. HPV, human papillomavirus.

All 64 HPV 52‐positive samples were sequenced across the complete E6 gene (nt 10−548) and compared to the HPV 52 E6 reference sequences. Our findings showed that 61% of samples (39 out of 64 samples) belonged to the A lineage and 39% of specimens (25 out of 60 samples) were classified with the B lineage. Among samples that were infected with the A lineage, sublineage A1 was dominant as it was found in 32 out of 39 samples and the remaining of them belonged to the A2 sublineage (Figure 1). Both the C and D lineages were not found in this study (Table 1).

**Table 1:** HPV 52 sublineages identified in normal and premalignant/malignant of cervical samples from Iranian women.

Lineage	Accession number of references	Nucleotide substitutions patterns	Patterns of amino acid changes	Nucleic acid substitutions																		Studied groups		
				143	200	237	251	252	253	257	288	296	308	348	350	375	376	379	404	520	530	Normal (*N* = 44)	Premalignant /malignant (*N* = 20)	Total (*N* = 64)
				A	C	C	A	C	G	A	T	G	T	C	G	G	T	A	T	G	A	*N* (%)	*N* (%)	*N* (%)
A	X74481	‐	‐	‐	‐	‐	‐	‐	‐	‐	‐	‐	‐	‐	‐	‐	‐	‐	‐	‐	‐	19 (43.3)	10 (50.0)	29 (45.3)
	JQ293067	A143C/G375T	E14D/V92L	C	‐	‐	‐	‐	‐	‐	‐	‐	‐	‐	‐	T	‐	‐	‐	‐	‐	1 (2.2)	‐	1 (1.6)
	‐	A257G/T288C/G520A	I52M/C63R	‐	‐	‐	‐	‐	‐	G	C	‐	‐	‐	‐	‐	‐	‐	‐	A	‐	1 (2.2)	‐	1 (1.6)
	HQ537737	T404C	‐	‐	‐	‐	‐	‐	‐	‐	‐	‐	‐	‐	‐	‐	‐	‐	C	‐	‐	‐	1 (5.0)	1 (1.6)
	HQ537739	A251G/A379G	K93R	‐	‐	‐	G	‐	‐	‐	‐	‐	‐	‐	‐	‐	‐	G	‐	‐	‐	6 (13.7)	1 (5.0)	7 (10.9)
B	HQ537740	G350T/A379G	K93R	‐	‐	‐	‐	‐	‐	‐	‐	‐	‐	‐	T	‐	‐	G	‐	‐	‐	1 (2.2)	1 (5.0)	2 (3.1)
	KJ675771	G350T	‐	‐	‐	‐	‐	‐	‐	‐	‐	‐	‐	‐	T	‐	‐	‐	‐	‐	‐	15 (34.2)	7 (35.0)	22 (34.3)
	‐	G350T/T376C	V92A	‐	‐	‐	‐	‐	‐	‐	‐	‐	‐	‐	T	‐	C	‐	‐	‐	‐	1 (2.2)	‐	1 (1.6)
C	HQ537744	C252A/G253A/G296A/T308C/G350T/A530G	R51K/M65I	‐	‐	‐	‐	A	A	‐	‐	A	C	‐	T	‐	‐	‐	‐	‐	G	‐	‐	‐
	HQ537746	C348G/G350T/A530G	L83V	‐	‐	‐	‐	‐	‐	‐	‐	‐	‐	G	T	‐	‐	‐	‐	‐	G	‐	‐	‐
D	HQ537748	C200T/C237T/G350T	‐	‐	T	T	‐	‐	‐	‐	‐	‐	‐	‐	T	‐	‐	‐	‐	‐	‐	‐	‐	‐

					Amino acid changes																			64 (100)
					E14D	‐	‐	‐	R51K	I52M	C63R	M65I	‐	L83V	‐	V92L	V92A	K93R	‐	‐	‐			

Abbreviation: HPV, human papillomavirus.

Sequence analysis of 64 specimens found that the nt changes happened in 35 samples in comparison to the prototype sequence (X74481). These substitutions occurred in 10 nucleic acid positions, including A143C, A251G, A257G, T288C, G350T, G375T, T376C, A379G, T404C, and G520A (Table 1). Among which, six nt substitutions at the positions of A143C, A257G, T288C, G375T, T376C, and A379G were non‐synonymous and led to amino acid substitutions at amino acid positions of E14D, I52M, C63R, V92L, V92A, and K93R, respectively. The remaining four nt changes, including A251G, G350T, T404C, and G520A, were silent mutations. Among six amino acid substitutions that were found in this study, the amino acid change K93R was more prevalent than other changes as was shown in nine out of 64 samples (14.1%). The remaining five amino acid changes were found only in one sample.

As shown in Table 1, eight different nt substitution patterns, including no change; A143C/G375T, A257G/T288C/G520A, T404C, A251G/A379G, G350T/A379G, G350T, and G350T/T376C were found in this study. Five different amino acid change patterns, including no amino acid change; E14D/V92L, I52M/C63R, V92A, and K93R were found, among which no amino acid change pattern was more prevalent, as was observed in 81.2% of samples.

As indicated in Table 2, stratification by cytological/histological types has shown that the A lineage was more common in normal and premalignant/malignant groups as it was found in 61.3% and 60% of samples, respectively. The difference that was observed between the two groups was not found to be statistically significant (*p* > 0.05). According to age groups, no statistically significant differences were observed between the two studied groups (*p* > 0.05) (Table 2).

**Table 2:** The frequency of HPV 52 lineages stratified by histology/cytology status or age in cervical samples of Iranian women.

Variables		Lineage A *N* (%)	Lineage B *N* (%)	Total *N* (%)	*p* Value (two‐tail)	Odds ratio (95% CI)
Histology/cytology status						
	Normal	27 (61.3)	17 (38.7)	44 (100)	0.918	1.06 (0.36−3.1)
	Premalignant/malignant	12 (60.0)	8 (40.0)	20 (100)		
Age (years)						
	<30	13 (56.5)	10 (43.5)	23 (100)	0.590	0.75 (0.26−2.1)
	≥30	26 (63.4)	15 (36.6)	41 (100)		

Abbreviations: CI, confidence interval; HPV, human papillomavirus.

## 4 DISCUSSION

It is shown that the distribution of HPV 16 and 18 lineages and sublineages can be different geographically as a result of the evolution associated with the population ethnicity.^23^, ^24^, ^25^, ^26^ However, the geographic relations between the lineages and sublineages of other HPV types remain to be clarified. It also considers that distinctive variants of each HR‐HPV type show differences in infectivity, long‐term persistence, and progression to premalignant and malignant lesions.^12^ In this regard, the present study investigated the sequence variations of the E6 gene of HPV 52 to characterize the prevalence of HPV 52 lineages and sublineages in normal and premalignant/malignant samples of the cervix of Iranian women.

The results of this study found that 61% and 39% of samples belonged to the A and B lineages, respectively, and two lineages of C and D were not detected. Among samples that were infected with the A lineage, sublineage A1 was dominant as it was found in 82% of samples and the remaining 18% belonged to the A2 sublineage (Figure 1 and Table 1). This result is almost compatible with several studies in the world. The result of one study from Canada indicated that the A lineage is prominent and other lineages, including B, C, and D, were found at lower frequencies.^27^ Another study in America showed the A lineage was more common than other lineages as 93.1% of samples were infected with the A lineage while the B, C, and D lineages were detected in 4.7%, 1.3%, and 0.9% of cases, respectively.^16^ In contrast to Western countries where the A lineage is prominent, several studies have shown that the B lineage is more prevalent in countries of East Asia. In Korea, the B lineage was detected in 86.8% of samples, followed by the C (6.6%), A (5.5%), and D (1.1%) lineages.^28^ One study from Japan, the Philippines, and Vietnam indicated that the B lineage was prominent in these countries. Although the B lineage was found to be dominant, the frequency of the B lineage was shown to be different as it was detected between 92.3%, 73.7%, and 62.5% of samples from Japan, the Philippines, and Vietnam, respectively. Other lineages that have been found in these countries were as follows: 5.1% of the A lineage and 2.6% of the D lineage in Japan; 15.8% of the A lineage, 2.6% of the C lineage, and 2.6% of the D lineage in Vietnam; and 34.4% of the A lineage and 3.1% of the D lineage in the Philippines.^29^ In Taiwan, the B lineage was reported in 88.2% of samples and the C and A lineages were found in 11.1% and 0.7% of cases, respectively.^30^ The result of a global study from four continents America, Europe, Africa, and Asia, has shown that the distribution of HPV 52 lineages is associated with ethnicity. In America, Europe, and Africa, the A lineage was dominant, while in Asia the B lineage was common. In America, the A, B, C, and D lineages were found in 83.7%, 5.5%, 9.1%, and 1.8% of samples, respectively. In Europe, the A lineage was detected in 96.8% of cases, followed by the C (2.1%) and B (1.1%) lineages. In Africa, two lineages A and D were reported in 78.6% and 21.4% of samples, respectively. In Asia, however, the B lineage was found in 89% of cases, followed by the A (5.5%), C (4.3%), and D (1.1%) lineages.^31^ Looking at the distribution of various sublineages, it was revealed that the A1 sublineage was more common than the A2 sublineage in America, Europe, and Africa, while in Asia the B2 sublineage was prominent.^31^ It is suggested that HPV variants may have coevolved with archaic hominins and spread across the globe through host interbreeding and gene flow. The coevolution of HPV variants with humans is documented for at least HPV 16 and 58. The differential coevolution of HPV 16 and HPV 58 variants with closely related ancestral human populations together with the introgression of specific archaic alleles associated with keratinocyte differentiation and innate immunity into the human genomes might result in the dominance of a special lineage in the modern human ancestor population. This event might play an important role in the different distribution of HPV variants in the world.^32^, ^33^

Regarding the finding that lineage A of HPV 52 is prevalent in Iran, the E6 variation sequence analysis of other HPV types including HPV 16, 18, 31, 39, 45, and 56 in Iranian women showed that distinctive lineage/sublineages are dominant in this population as follows: for HPV 16 (lineage D), HPV 18 (sublineage A4), HPV 31 (lineage A), HPV 39 (sublineage A1), HPV 45 (lineage B), and HPV 56 (lineage B).^15^, ^18^, ^19^, ^20^, ^21^

In this study, no statistically significant difference was observed about distinct lineages between normal and premalignant/malignant groups, as the A lineage was common in both groups (Table 2). As no association was observed between HPV 52 variants and pathological stages, it is more likely that the low sample size does not allow us to find major differences. Compatible with our result, in one study, no association was found between the stage of disease and HPV 52 variants among Chinese women.^34^ However, amino acid changes may confer higher oncogenesis potential. Indeed, the amino acid substitution of K93R was reported to be more prevalent (97.8%) in premalignant and malignant cervical samples in Japanese women.^35^ One study from Canada revealed that K93R amino acid change significantly increased the risk of progression to CIN 2,3.^27^ To best understand the effect of amino acid substitutions on oncogenesis, in one study, four different plasmids of the HPV 52 E6 gene, including (1) prototype; (2) amino acid change at K93R (V1); (3) amino acid substitutions at E14D and V92L (V2); and (4) amino acid changes at K93R and N122K (V3) were constructed and the potential of carcinogenesis was evaluated. Interestingly, the result revealed that the V1 variant has shown stronger transforming activity. The V1 variant was also found to promote greater cell migration ability than three other plasmids. Concerning these findings, it is suggested that K93R amino acid substitution probably promotes more efficient downregulation of E‐cadherin, leading to increased cell migration ability.^36^ To support this finding, it is worth mentioning that HPV 52 is more prevalent in East Asia than in other parts of the world and this type ranked as the fourth most common HPV genotype in this area.^37^, ^38^ Regarding these points, K93R amino acid substitution is specific to the B lineage and this lineage is more prevalent in East Asia where HPV 52 has high frequency, it should be considered that the K93R change probably enhances carcinogenesis of HPV 52.

Interestingly, it has been suggested that race‐associated differences in the persistency of HPV 52 lineages can lead to a less effective T‐cell response in clearing infections due to the modulation of host cellular immunity. The modulation of cellular immune responses can be affected by the host factors including HLA molecules, viral factors such as LCR, E6, E7, and L1 sequence variability, or both of them.^26^, ^39^, ^40^ Indeed, it is suggested that the natural variation in L1 antigenicity in distinctive HPV 52 variants can impact the neutralization by nonavalent HPV vaccine (Gardasil®9) antibodies. The HPV52 D variant showed >fourfold lower sensitivities in comparison to their consensus A/A1 variant. This means that the D lineage should be considered a distinct serotype.^41^

In conclusion, our findings revealed that the A lineage, the sublineage of A1, and the B lineage are circulating in Iran. Nevertheless, further studies with larger sample sizes are needed to estimate the pathogenicity risk of HPV 52 variants in Iran. To better understand the association between HPV variants and the genetic background, it is highly recommended that the characterization of HLA molecules be considered in future studies. The integration status of samples will be investigated in the future.

## AUTHOR CONTRIBUTIONS

**Parvin Jalali‐Alhosseini**: Formal analysis; methodology; validation. **Zabihollah Shoja**: Data curation; investigation; project administration; software; visualization; writing—original draft. **Somayeh Jalilvand**: Conceptualization; funding acquisition; resources; supervision; validation; writing—review and editing. All authors have read and approved the final version of the manuscript.

## CONFLICT OF INTEREST STATEMENT

The authors declare no conflict of Interest.

## ETHICS STATEMENT

Our research was conducted ethically by the World Medical Association Declaration of Helsinki. We declare that informed consent was obtained from all study subjects and the study was approved by the local ethical committee of Tehran University of Medical Sciences (IR.TUMS.SPH.REC.1399.253).

## TRANSPARENCY STATEMENT

The lead author Somayeh Jalilvand affirms that this manuscript is an honest, accurate, and transparent account of the study being reported; that no important aspects of the study have been omitted; and that any discrepancies from the study as planned (and, if relevant, registered) have been explained.

## Data Availability

The sequences deposited in NCBI with the following accession numbers: OL627421‐OL627480. Corresponding author had full access to all of the data in this study and take complete responsibility for the integrity of the data and the accuracy of the data analysis.
